# Supplementary material for: Somatic mutation distribution across tumour cohorts provides a signal for positive selection in cancer
Source: Nat Commun. 2022 Nov 17;13:7023. doi: 10.1038/s41467-022-34746-z (PMC9671924; doi:10.1038/s41467-022-34746-z)
Supplement: Supplementary file 1 — Supplementary Information [file 41467_2022_34746_MOESM1_ESM.pdf]

# Boström, Larsson

# Supplementary appendix

## Table of Contents

***Supplementary Figure 1*** .......... **2**

***Supplementary Figure 2*** .......... **3**

***Supplementary Figure 3*** .......... **4**

***Supplementary Figure 4*** .......... **5**

***Supplementary Figure 5*** .......... **6**

***Supplementary Figure 6*** .......... **7**

***Supplementary Figure 7*** .......... **8**

***Supplementary Figure 8*** .......... **9**

***Supplementary Figure 9*** .......... **10**

***Supplementary Figure 10*** .......... **11**

***Supplementary Figure 11*** .......... **12**

***Supplementary Figure 12*** .......... **13**

***Supplementary Figure 13*** .......... **14**

***References*** .......... **15**

# Supplementary Figure 1

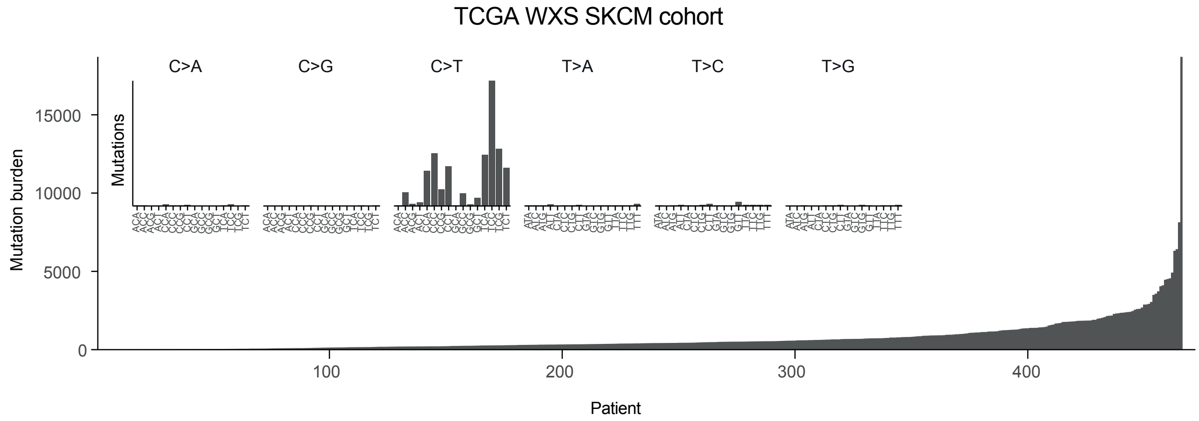


**SKCM cohort summary.** Number of mutations in each patient in the TCGA SKCM cohort. The mutations exhibited an overall trinucleotide mutation pattern in agreement with the known signature of UV light, dominated by C>T transitions in dipyrimidine contexts (inset). TCGA, The Cancer Genome Atlas; WXS, whole-exome sequencing; SKCM, cutaneous melanoma. Source data are provided as a Source Data file.

# Supplementary Figure 2

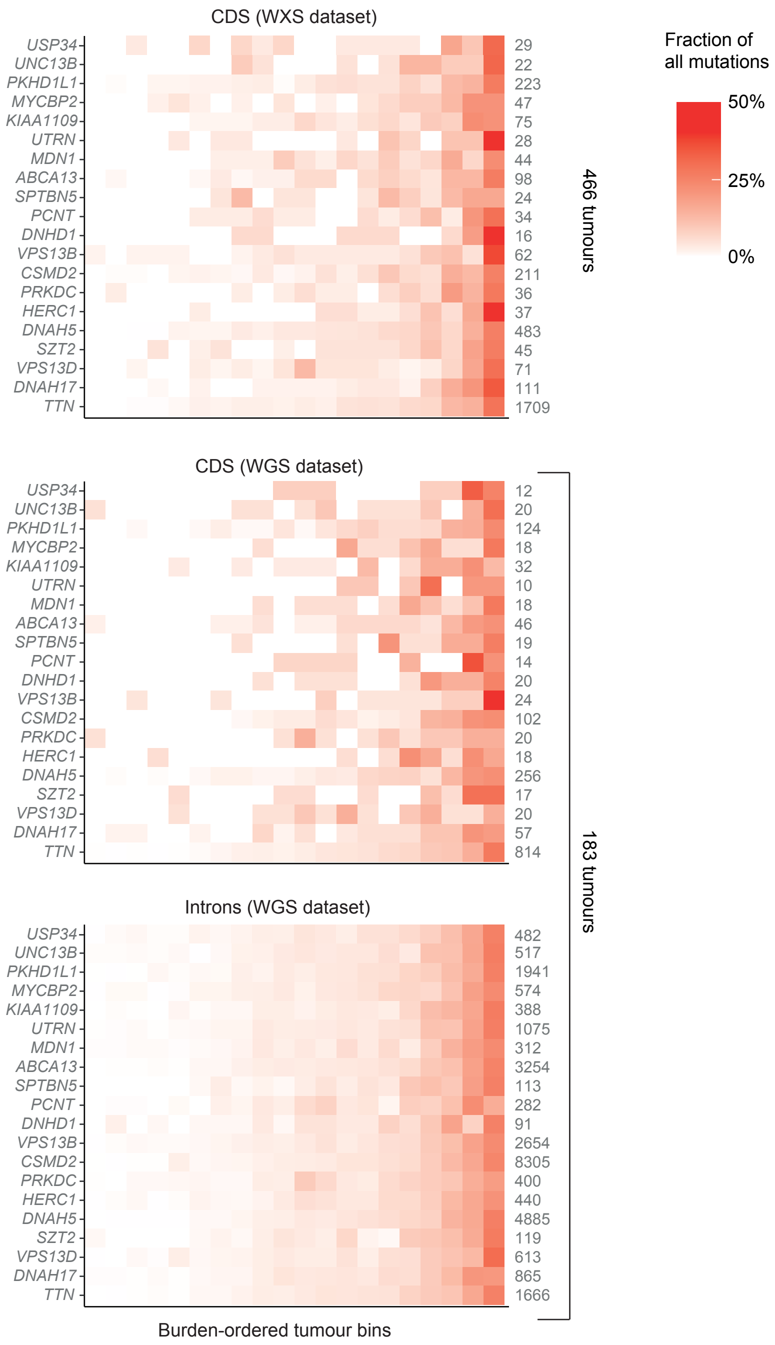


**Heatmap of mutations in tumours ordered by mutation burden and binned, for non-cancer genes.** 20 non-CGC genes were randomly selected from genes with CDSs and introns of at least 10 kb, in order to make sure there were enough mutations to make a clear heatmap showing the passenger pattern. The mutation data and procedure are the same as in **Fig. 2 b-c**. CDS, coding sequence; WXS, whole-exome sequencing; WGS, whole-genome sequencing. Source data are provided as a Source Data file.

# Supplementary Figure 3

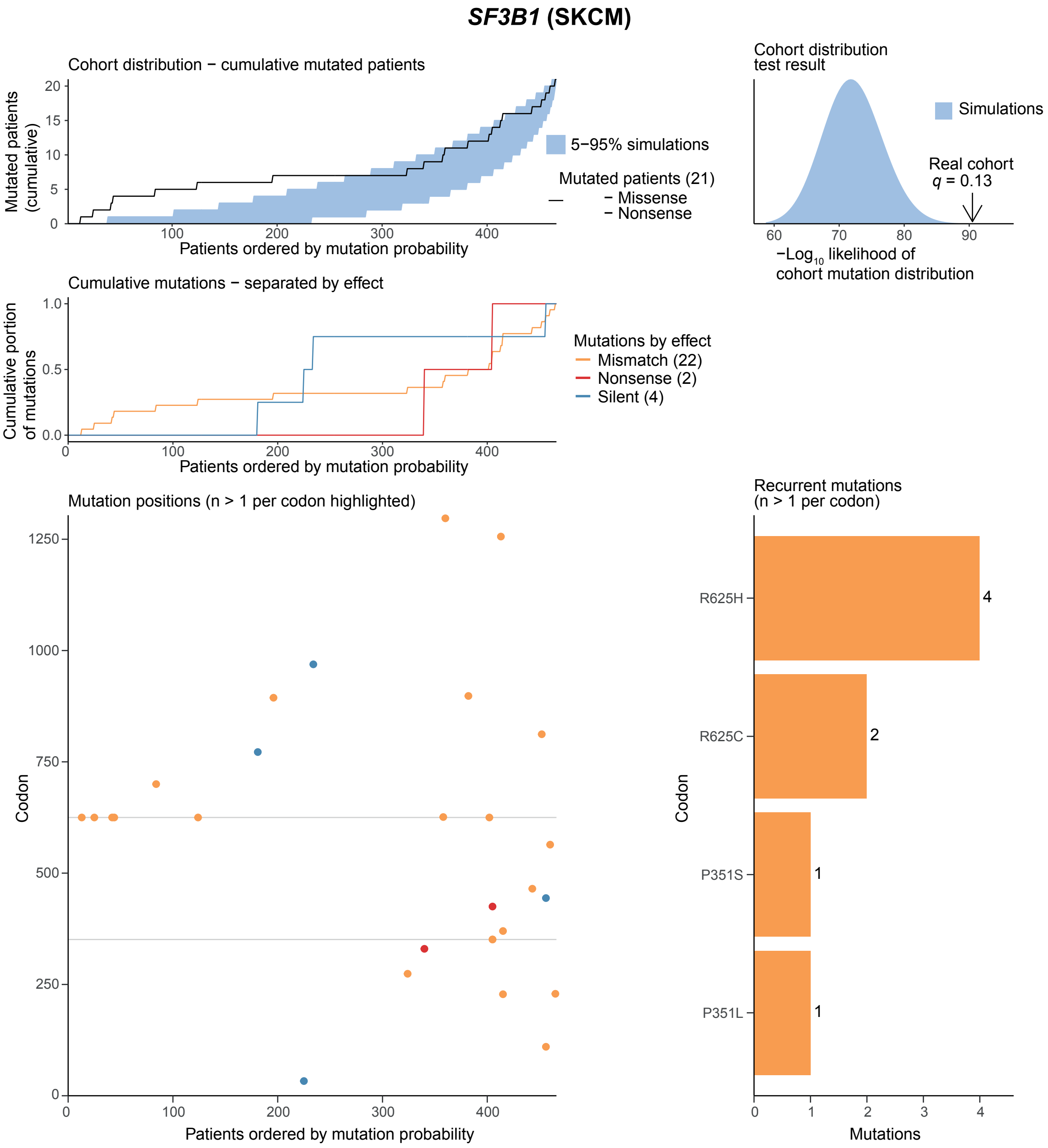


**Cohort and positional distributions for somatic mutations in *SF3B1* in SKCM. Top**: CMT plot, as described in **Fig. 1**. *q*-value, false discovery rate. **Middle:** The cumulative fraction of all the mutations for each effect (missense, nonsense, synonymous) vs. tumours ordered by mutation probability. **Bottom:** Locations of mutations in the gene vs burden-ordered tumours, with recurrent mutations marked with horizontal lines and summarised in the adjacent bar plot. SKCM, cutaneous melanoma ($n$ = 466 tumours). Source data are provided as a Source Data file.

# Supplementary Figure 4

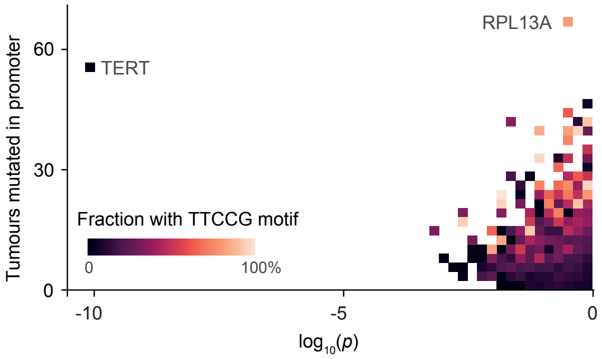


**Resilience to TTCCG-related model flaws in promoters in melanoma using combined methods.** Same as in **Fig. 3a**, but with the more conservative *p*-value (uncorrected) from the two methods (SEISMIC and ActiveDriverWGS) used for each promoter. The result is a better separation between the *TERT* promoter, a true driver, and the other promoters than either method yielded on their own. Source data are provided as a Source Data file.

# Supplementary Figure 5

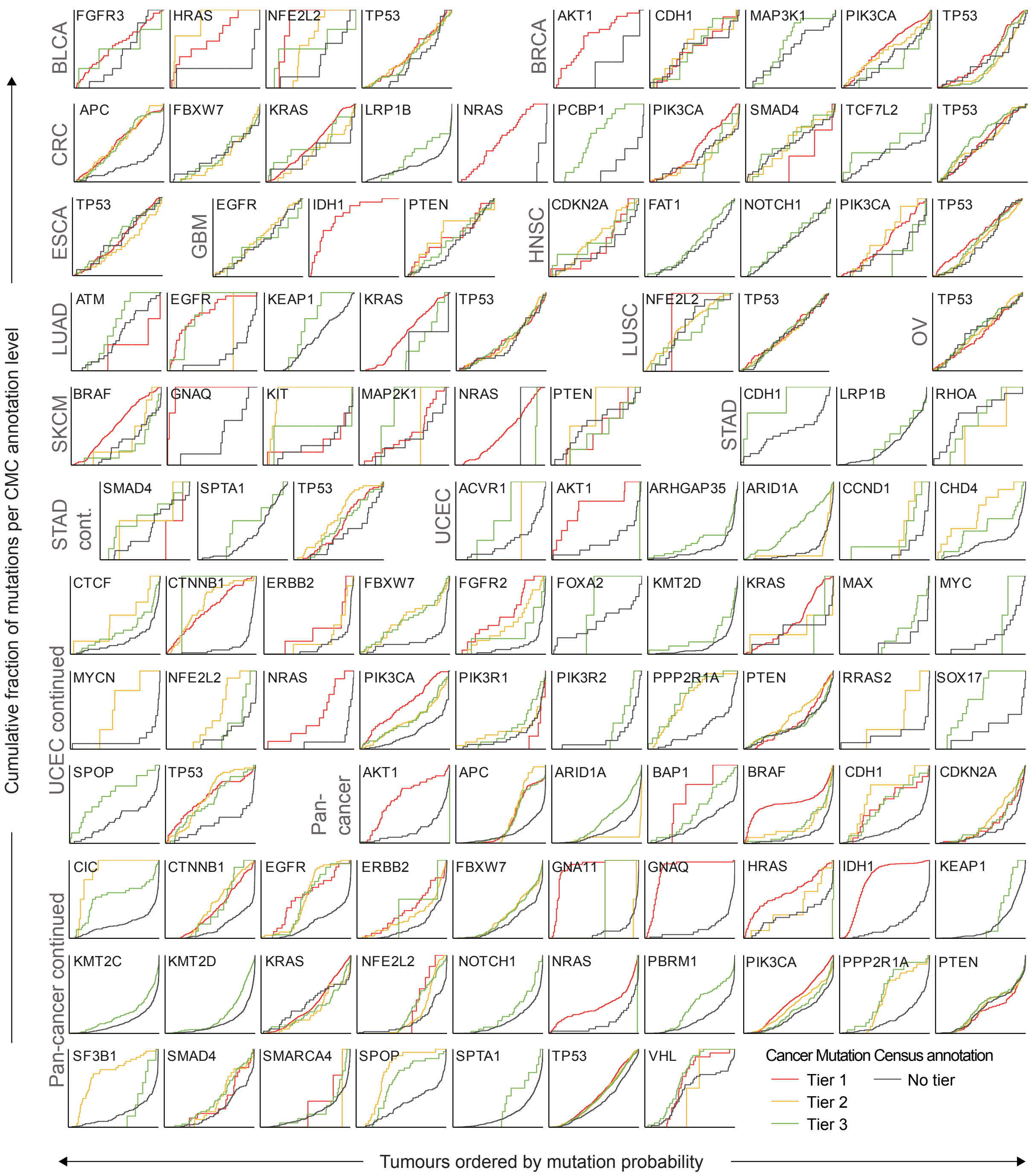


**Cumulative fraction of mutations with different levels of support in the Cancer Mutation Census.** All genes in **Fig. 4a** where at least one mutation is annotated as having tier 1, 2, or 3 support of being a cancer driver in CMC are included. In general, known and putative driver mutations skew toward low-probability patients, as expected for mutations under positive selection. Source data are provided in Supplementary Data 7.

# Supplementary Figure 6

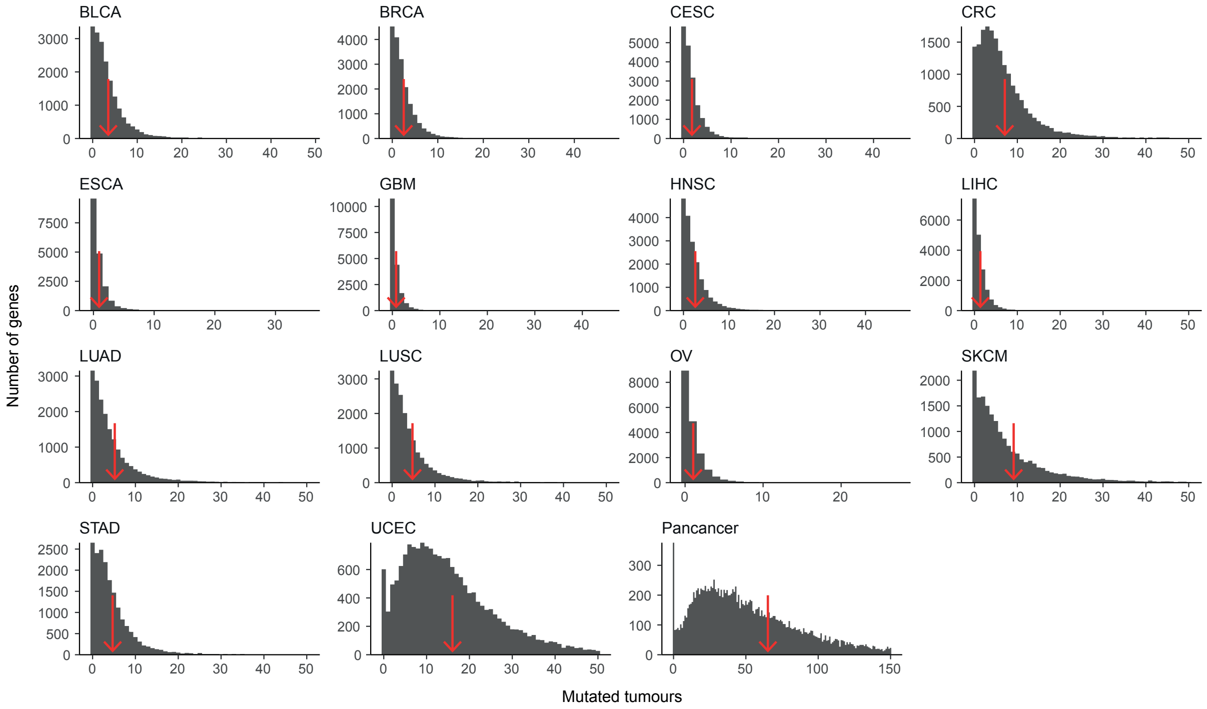


**Histogram of the number of mutated tumours per gene in each cancer type analysed in Fig. 4a.** Red lines indicate the average number of mutated tumours for all genes. UCEC had the highest average number of mutated tumours (apart from pan-cancer), and is also the cancer type where the most genes were uncovered with SEISMIC, in agreement with high burden being beneficial for the statistical power. BLCA, bladder carcinoma; BRCA, breast carcinoma; CESC, cervical carcinoma; CRC, colorectal carcinoma; ESCA, esophageal carcinoma; GBM, glioblastoma; HNSC, head and neck carcinoma; LIHC, liver hepatocellular carcinoma; LUAD, lung adenocarcinoma; LUSC, lung squamous cell carcinoma; OV, ovarian adenocarcinoma; SKCM, cutaneous melanoma; STAD, stomach adenocarcinoma; UCEC, endometrial carcinoma. Source data are provided as a Source Data file.

# Supplementary Figure 7

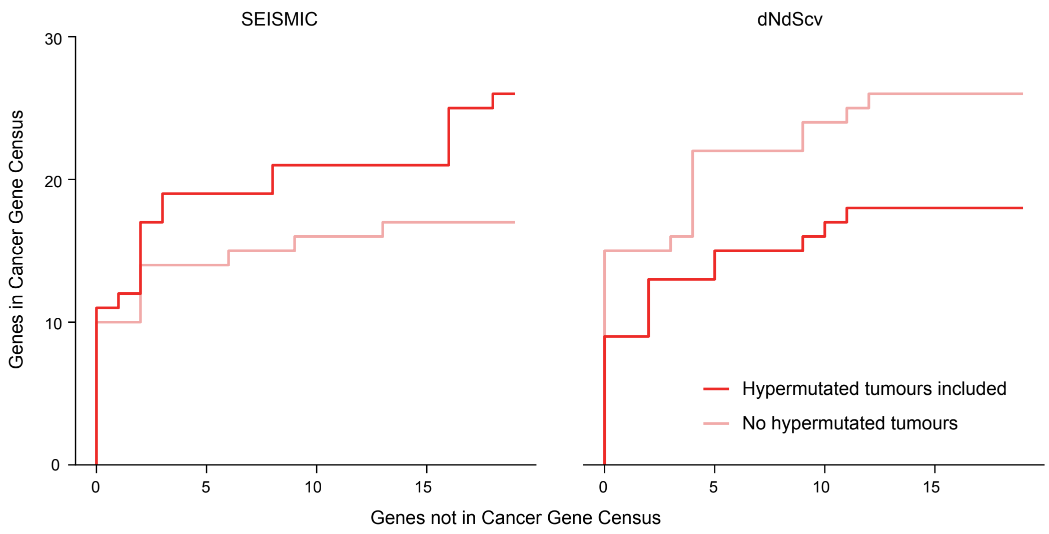


**Impact of including hypermutated tumours on Cancer Gene Census gene enrichment.** Cumulative plot showing enrichment of canonical cancer genes as in **Fig. 4b** for the UCEC cohort, with and without hypermutated tumours, for SEISMIC and dNdScv. Tumours with at least 2000 mutations were classified as hypermutated, constituting 61 out of 529 total tumours. dNdScv parameters to disable hypermutation filtering are described in **Methods**. Source data are provided in Supplementary Data 1, 8-10.

# Supplementary Figure 8

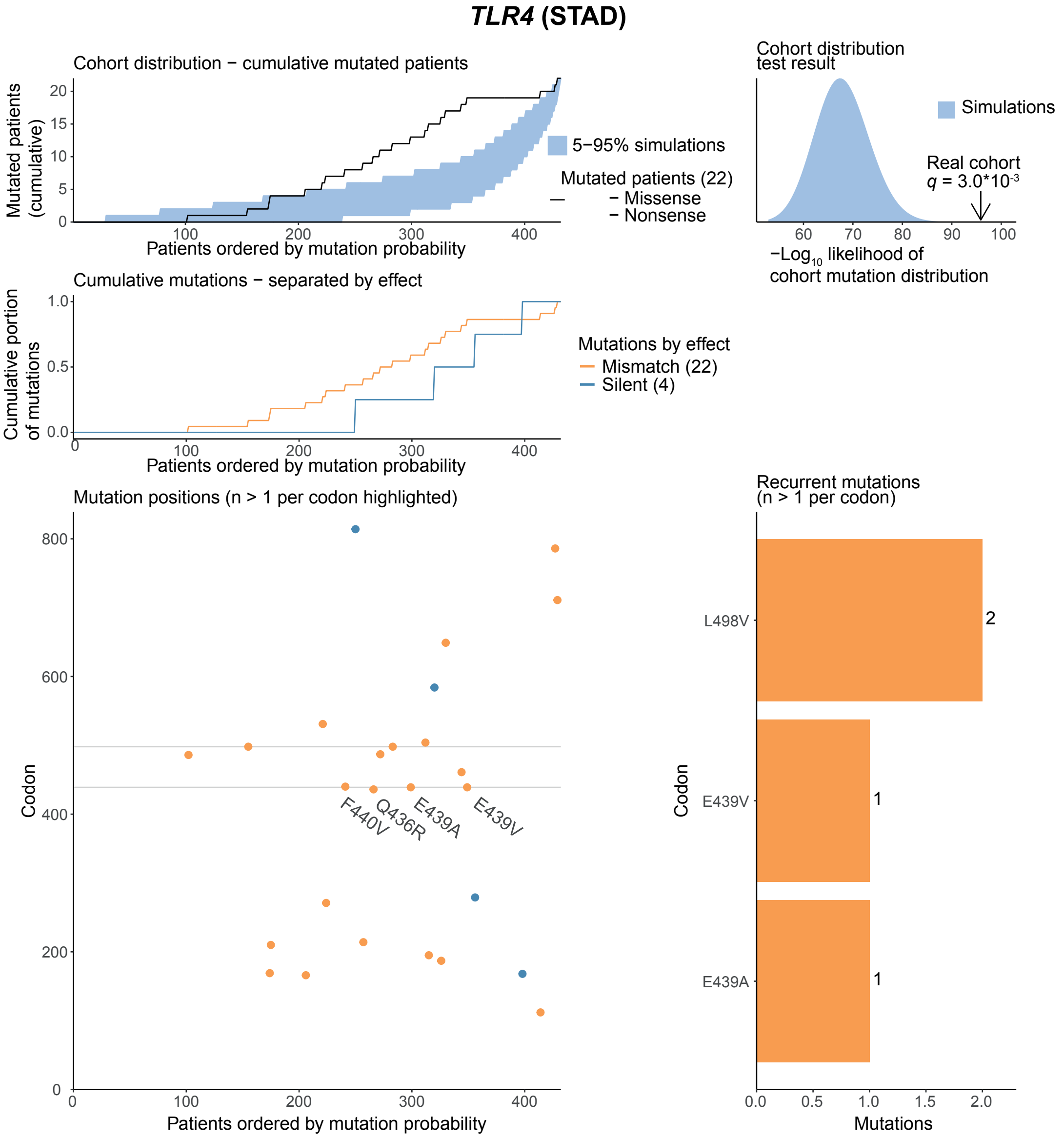


**Cohort and positional distributions for somatic mutations in *TLR4* in STAD. Top**: CMT plot, as described in **Fig. 1**. $q$-value, false discovery rate. **Middle:** The cumulative fraction of all the mutations for each effect (missense, nonsense, synonymous) vs. tumours ordered by mutation probability. **Bottom:** Locations of mutations in the gene vs burden-ordered tumours, with recurrent mutations marked with horizontal lines and summarised in the adjacent bar plot. STAD, stomach adenocarcinoma ($n$ = 432 tumours). Source data are provided as a Source Data file.

# Supplementary Figure 9

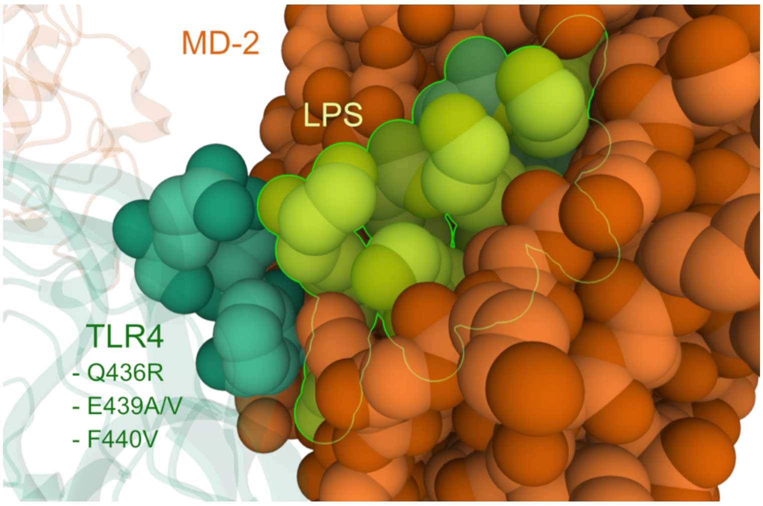


**Interface between TLR4, MD-2, and LPS.** Screenshot of Mol* Viewer[1] on rcsb.org, showing PDB ID 3FXI [2], with LPS (lipopolysaccharide), MD-2 (myeloid differentiation 2) and 3 sites with clustered mutations in TLR4 in spacefill representation.

# Supplementary Figure 10

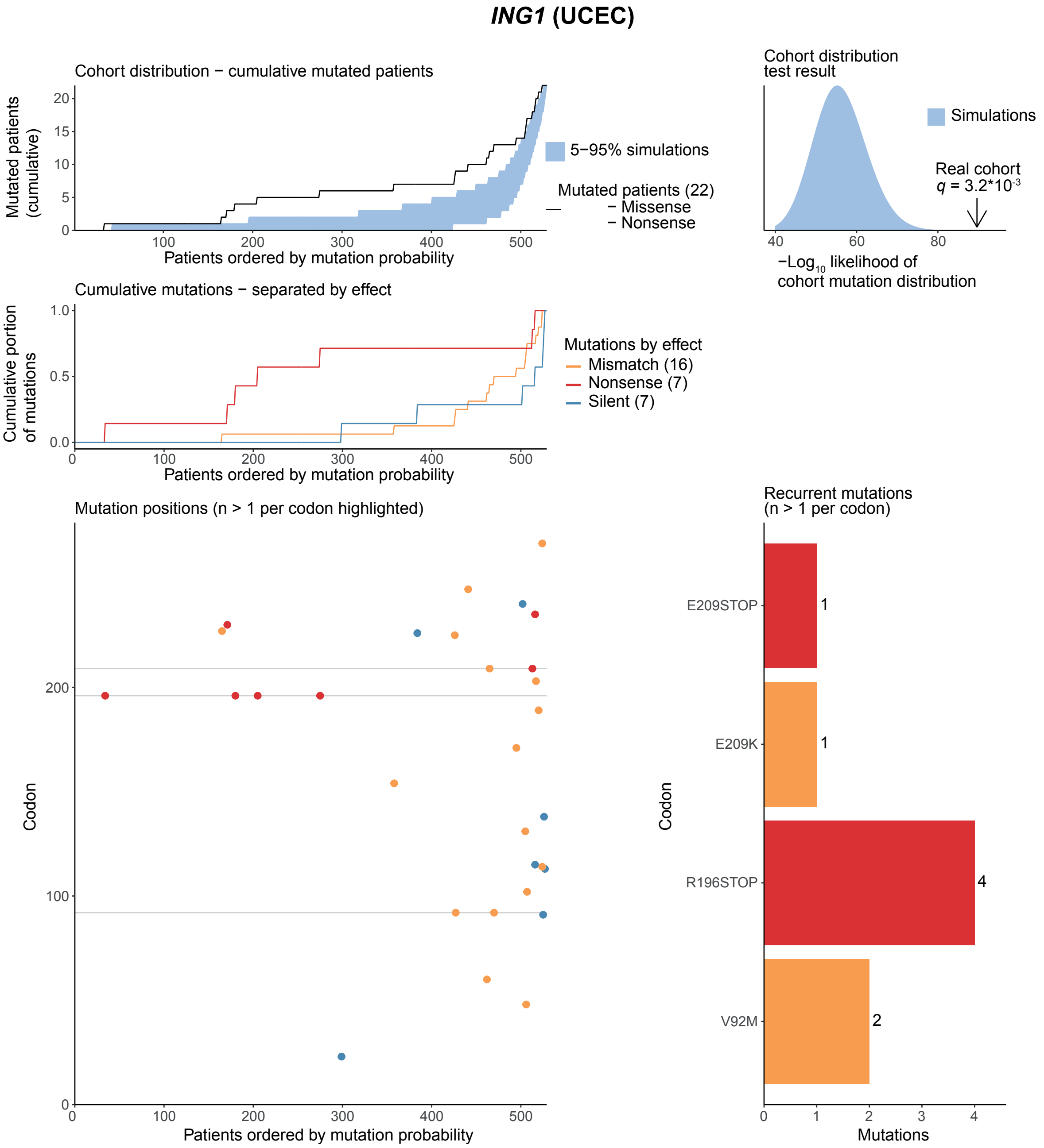


**Cohort and positional distributions for somatic mutations in *ING1* in UCEC. Top**: CMT plot, as described in **Fig. 1**. $q$-value, false discovery rate. **Middle:** The cumulative fraction of all the mutations for each effect (missense, nonsense, synonymous) vs. tumours ordered by mutation probability. **Bottom:** Locations of mutations in the gene vs burden-ordered tumours, with recurrent mutations marked with horizontal lines and summarised in the adjacent bar plot. UCEC, endometrial carcinoma ($n$ = 529 tumours). Source data are provided as a Source Data file.

# Supplementary Figure 11

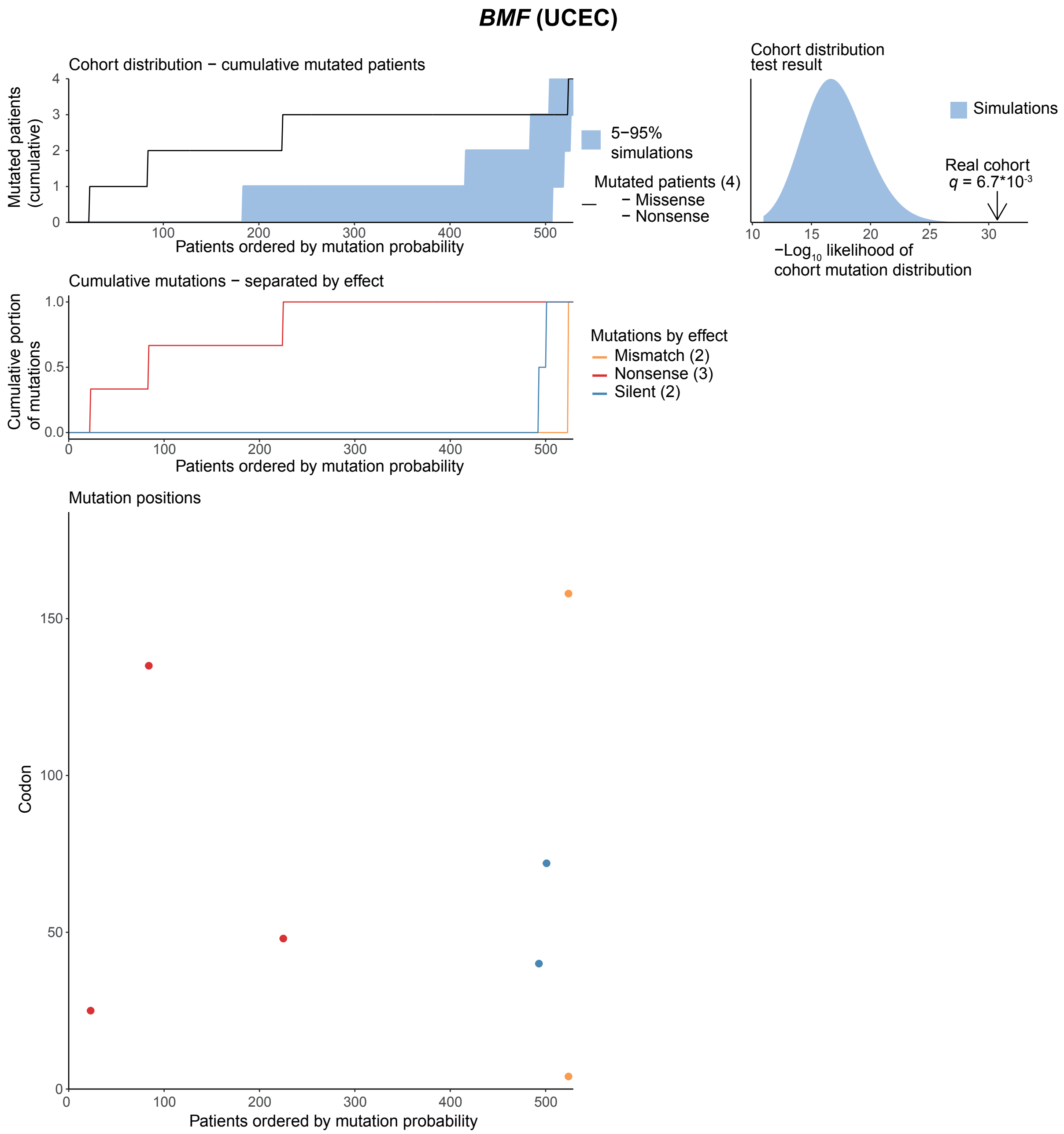


**Cohort and positional distributions for somatic mutations in *BMF4* in UCEC. Top**: CMT plot, as described in **Fig. 1**. *q*-value, false discovery rate. **Middle:** The cumulative fraction of all the mutations in *BMF4* for each effect (missense, nonsense, synonymous) vs. tumours ordered by mutation probability. **Bottom:** Locations of mutations in the gene vs burden-ordered tumours. UCEC, endometrial carcinoma ($n$ = 529 tumours). Source data are provided as a Source Data file.

# Supplementary Figure 12

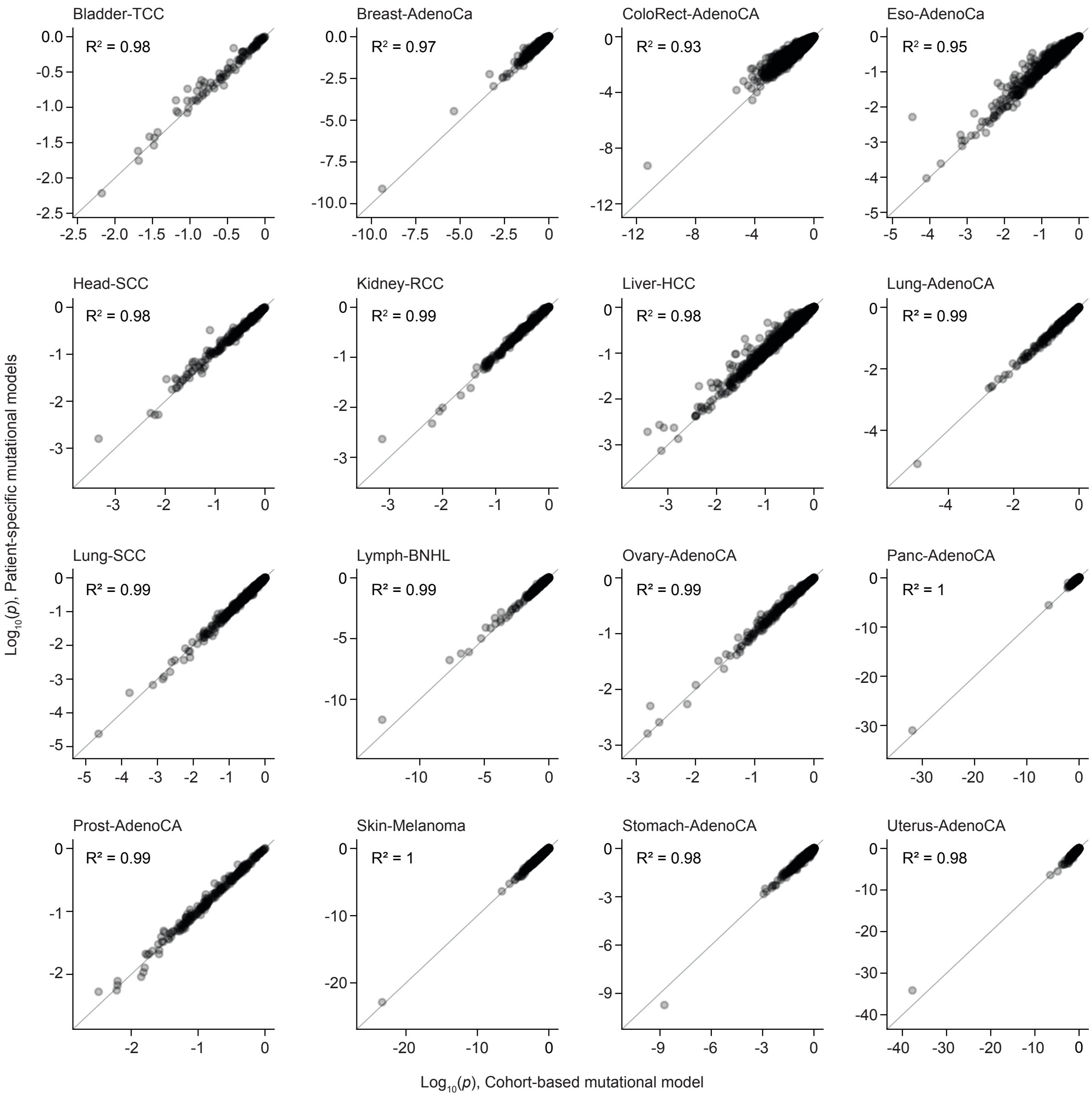


**Correlation between SEISMIC results with patient-specific and cohort-based mutational models.** SEISMIC was run on PCAWG WGS mutation data for cancer types with more than 100 genes that harbour mutations in at least 3 tumours, once each with cohort-based and patient-specific mutational models. The correlation between the results is shown here with uncorrected SEISMIC *p*-values. Diagonal lines show identical *p*-values for both models. $R^2$ values indicate the correlation (Pearson) between the results using these two models. Source data are provided as a Source Data file.

# Supplementary Figure 13

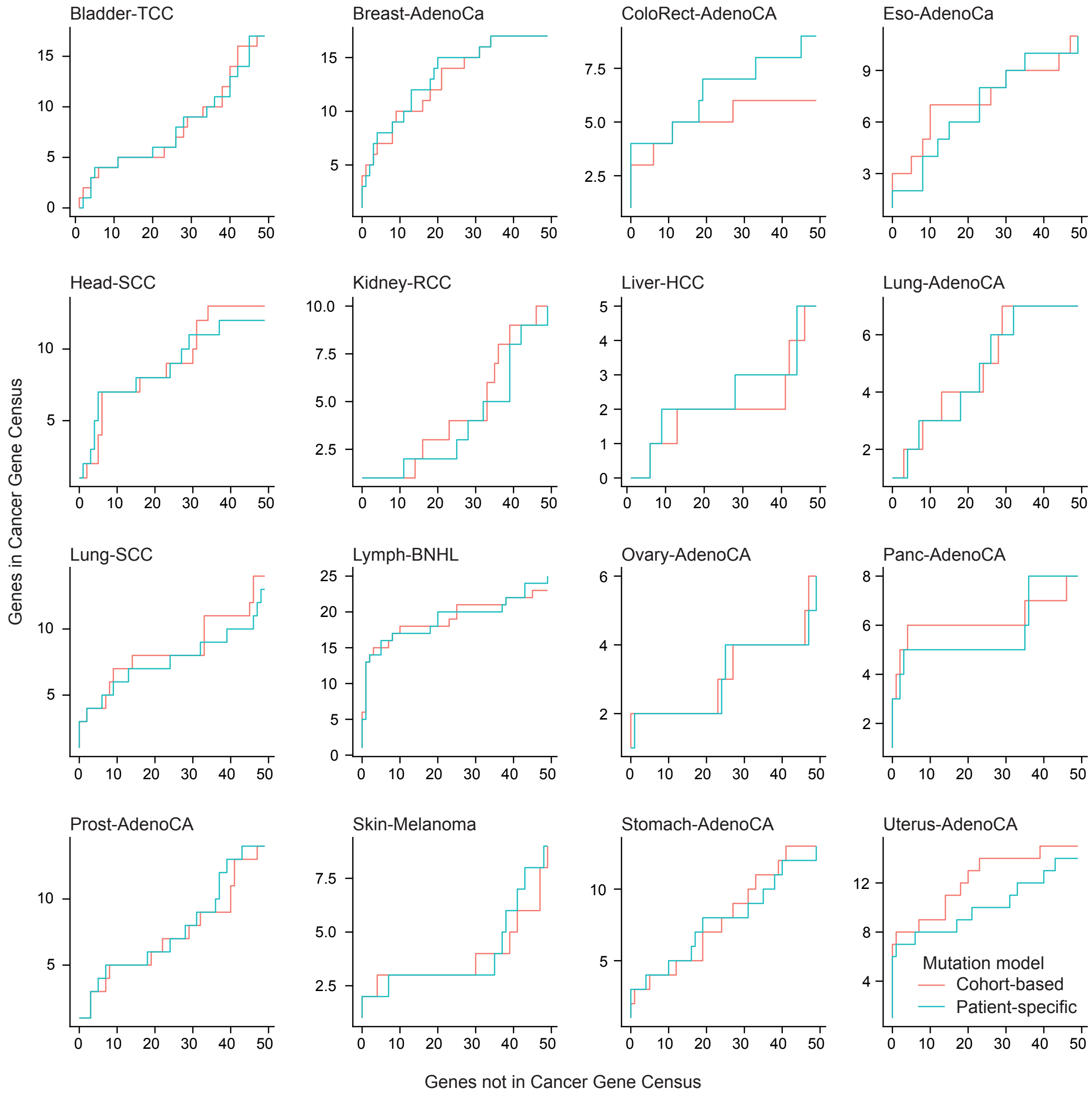


**Enrichment of canonical cancer genes in SEISMIC results for patient-specific and cohort-based mutational models.** SEISMIC was run on PCAWG WGS mutation data for cancer types with more than 100 genes that harbour mutations in at least 3 tumours. The cumulative number of genes present in the Cancer Gene Census, or not, is plotted by order of significance in these results. Source data are provided in Supplementary Data 14-15.

# References

1 Sehnal, D. *et al.* Mol* Viewer: modern web app for 3D visualization and analysis of large biomolecular structures. *Nucleic Acids Research* **49**, W431-W437 (2021). https://doi.org:10.1093/nar/gkab314

2 Park, B. S. *et al.* The structural basis of lipopolysaccharide recognition by the TLR4-MD-2 complex. *Nature* **458**, 1191-1195 (2009). https://doi.org:10.1038/nature07830
