# Supplementary material for: Somatic mutation distribution across tumour cohorts provides a signal for positive selection in cancer
Source: Nat Commun. 2022 Nov 17;13:7023. doi: 10.1038/s41467-022-34746-z (PMC9671924; doi:10.1038/s41467-022-34746-z)
Supplement: Supplementary file 3 — Description of Additional Supplementary Files [file 41467_2022_34746_MOESM3_ESM.pdf]

Description of Additional Supplementary Files

File Name: Supplementary Data 1
Description: SEISMIC results in CDS regions, TCGA WXS data

File Name: Supplementary Data 2
Description: dNdScv results in CDS regions, TCGA WXS SKCM

File Name: Supplementary Data 3
Description: MutSigCV results in CDS regions, TCGA WXS SKCM

File Name: Supplementary Data 4
Description: SEISMIC analysis of promoters in melanoma

File Name: Supplementary Data 5
Description: ActiveDriverWGS results from analysis of promoters in melanoma

File Name: Supplementary Data 6
Description: SEISMIC analysis of promoters in PCAWG WGS data

File Name: Supplementary Data 7
Description: Mutations in all genes in Figure 4a

File Name: Supplementary Data 8
Description: SEISMIC analysis of CDS regions in TCGA UCEC data, limited to non-hypermutated tumours (results including hypermutated tumours are found in Supplementary Data 1). Tumours with at least 2000 mutations were classified as hypermutated, excluding 61 tumours from the full cohort of 529 UCEC tumours.

File Name: Supplementary Data 9
Description: dNdScv analysis of CDS regions in TCGA UCEC data, limited to non-hypermutated tumours. Tumours with at least 2000 mutations were classified as hypermutated, excluding 61 tumours from the full cohort of 529 UCEC tumours. dNdScv run with max_muts_per_gene_per_sample = Inf, max_coding_muts_per_sample = Inf to avoid internal filtering of hypermutated tumours.

File Name: Supplementary Data 10
Description: dNdScv analysis of CDS regions in TCGA UCEC data, including hypermutated tumours. dNdScv run with max_muts_per_gene_per_sample = Inf, max_coding_muts_per_sample = Inf to avoid internal filtering of hypermutated tumours. dNdScv run with max_muts_per_gene_per_sample = Inf, max_coding_muts_per_sample = Inf to avoid internal filtering of hypermutated tumours.

File Name: Supplementary Data 11
Description: MutPanning results in CDS regions, TCGA WXS UCEC. Genes "1-Mar" and "2-Mar" in MutPanning output corrected to MARCHF1 and MARCHF2.

File Name: Supplementary Data 12
Description: dNdScv results in CDS regions, TCGA WXS UCEC

File Name: Supplementary Data 13
Description: MutSigCV results in CDS regions, TCGA WXS UCEC

File Name: Supplementary Data 14
Description: SEISMIC analysis of CDS regions in PCAWG WGS data using patient-specific mutational models

File Name: Supplementary Data 15
Description: SEISMIC analysis of CDS regions in PCAWG WGS data using cohort-based mutational models
