# Supplementary material for: Somatic mutation distribution across tumour cohorts provides a signal for positive selection in cancer
Source: Nat Commun. 2022 Nov 17;13:7023. doi: 10.1038/s41467-022-34746-z (PMC9671924; doi:10.1038/s41467-022-34746-z)
Supplement: Supplementary file 19 — Reporting Summary [file 41467_2022_34746_MOESM19_ESM.pdf]

nature portfolio

Corresponding author(s): Larsson

Last updated by author(s): Oct 26, 2022

Reporting Summary

Nature Portfolio wishes to improve the reproducibility of the work that we publish. This form provides structure for consistency and transparency in reporting. For further information on Nature Portfolio policies, see our Editorial Policies and the Editorial Policy Checklist.

Statistics

For all statistical analyses, confirm that the following items are present in the figure legend, table legend, main text, or Methods section.

n/a	Confirmed	
☐	☒	The exact sample size (n) for each experimental group/condition, given as a discrete number and unit of measurement
☒	☐	A statement on whether measurements were taken from distinct samples or whether the same sample was measured repeatedly
☐	☒	The statistical test(s) used AND whether they are one- or two-sided<br>*Only common tests should be described solely by name; describe more complex techniques in the Methods section.*
☐	☒	A description of all covariates tested
☐	☒	A description of any assumptions or corrections, such as tests of normality and adjustment for multiple comparisons
☐	☒	A full description of the statistical parameters including central tendency (e.g. means) or other basic estimates (e.g. regression coefficient) AND variation (e.g. standard deviation) or associated estimates of uncertainty (e.g. confidence intervals)
☐	☒	For null hypothesis testing, the test statistic (e.g. F, t, r) with confidence intervals, effect sizes, degrees of freedom and P value noted<br>*Give P values as exact values whenever suitable.*
☒	☐	For Bayesian analysis, information on the choice of priors and Markov chain Monte Carlo settings
☒	☐	For hierarchical and complex designs, identification of the appropriate level for tests and full reporting of outcomes
☐	☒	Estimates of effect sizes (e.g. Cohen's d, Pearson's r), indicating how they were calculated

Our web collection on statistics for biologists contains articles on many of the points above.

Software and code

Policy information about availability of computer code

Data collection	No software was used for data collection
Data analysis	The SEISMIC software, implemented in R, is available on GitHub (https://github.com/larsson-lab/SEISMIC)<br>At this time, the following package versions have been used/tested in SEISMIC:<br>R (4.1.0), tidyverse (1.3.1), yaml (2.3.5), foreach (1.5.2), doSNOW (1.0.20), stringi (1.7.6), inline (0.3.19), Rcpp (1.0.8.3), fitdistrplus (1.1.8), data.table (1.14.2), plyranges (1.14.0), BSgenome.Hsapiens.UCSC.hg19 (1.4.3), BSgenome.Hsapiens.UCSC.hg38 (1.4.4), cowplot (1.1.1)<br><br>Other driver detection tools:<br>ActiveDriverWGS (1.1.1), dNdScv (0.0.1.0), MutSigCV (1.3.5), MutPanning (2)<br><br>Other:<br>UCSC LiftOver (no version number available), VarScan (2.3), samtools (0.1.18)

For manuscripts utilizing custom algorithms or software that are central to the research but not yet described in published literature, software must be made available to editors and reviewers. We strongly encourage code deposition in a community repository (e.g. GitHub). See the Nature Portfolio guidelines for submitting code & software for further information.

Data

Policy information about availability of data

All manuscripts must include a data availability statement. This statement should provide the following information, where applicable:

- Accession codes, unique identifiers, or web links for publicly available datasets
- A description of any restrictions on data availability
- For clinical datasets or third party data, please ensure that the statement adheres to our policy

TCGA WXS mutation calls were downloaded from the NCI GDC portal (https://portal.gdc.cancer.gov/). WGS-based melanoma mutation calls from AMGP, as well as publicly available WGS pan-cancer mutation calls from PCAWG, were attained from the ICGC database (https://dcc.icgc.org/). The restricted TCGA portion of the PCAWG dataset was downloaded via Bionimbus (https://bionimbus-pdc.opensciencedatacloud.org/). TCGA WGS melanoma mutation calls were based on alignments downloaded from cgHub (cgHub is no longer available; TCGA WGS data is now accessible through GDC). Researchers need to apply for access to TCGA WGS data to the TCGA Data Access Committee (DAC) via dbGaP (https://dbgap.ncbi.nlm.nih.gov). The study makes use of Cancer Gene Census (v96) and Cancer Mutation Census (v96) data, downloaded from https://cancer.sanger.ac.uk, as well as RefSeq gene definitions (GRCh37.p13 and GRCh38.p13), downloaded from the UCSC ftp server (https://genome.ucsc.edu/). The MD-2/LPS interface protein structure (PDB ID 3FXI) was accessed at RCSB Protein Data Bank (https://www.rcsb.org/). Source data are provided with this paper.

Field-specific reporting

Please select the one below that is the best fit for your research. If you are not sure, read the appropriate sections before making your selection.

[x] Life sciences [ ] Behavioural & social sciences [ ] Ecological, evolutionary & environmental sciences

For a reference copy of the document with all sections, see nature.com/documents/nr-reporting-summary-flat.pdf

Life sciences study design

All studies must disclose on these points even when the disclosure is negative.

Sample size	No statistical methods were used to decide sample (cohort) size, as this was determined by TCGA data availability.
Data exclusions	Hypermutated tumours and lowly mutated cancer types were excluded as detailed in Methods. These criteria were established during testing of the method. The exclusions were made for the following reasons:<br>- Hypermutated tumours that contain a very large portion of a cohort's mutations can have an unduly large impact on results.<br>- In lowly mutated cancer types, too few genes remain to be analysed due to SEISMIC's default requirement of at least 3 mutated tumours for a given gene. Additionally, remaining genes may be enriched for cancer genes, due to positive selection and excess mutations in these genes, which complicates assessment of the performance of the tool.
Replication	Results were generally not replicated. The main goal of the study was to establish a new method and to evaluate the performance of the method compared to existing approaches, which was done repeatedly in several TCGA cancer types. Replicating the results for all individual cancer types would be challenging due to limited availability of suitable large sequencing datasets. In the case of melanoma, the feasibility of the principle the underlies SEISMIC was demonstrated through visualization of cohort mutation distribution patterns for known cancer driver genes and passenger genes in two independent datasets.
Randomization	Randomization is not applicable for this study, as it doesn't involve comparisons between groups.
Blinding	The analyses were not blinded, as no treated/untreated or similar distinction can be made.

Reporting for specific materials, systems and methods

We require information from authors about some types of materials, experimental systems and methods used in many studies. Here, indicate whether each material, system or method listed is relevant to your study. If you are not sure if a list item applies to your research, read the appropriate section before selecting a response.

Materials & experimental systems

n/a	Involved in the study
[x]	[ ] Antibodies
[x]	[ ] Eukaryotic cell lines
[x]	[ ] Palaeontology and archaeology
[x]	[ ] Animals and other organisms
[x]	[ ] Human research participants
[x]	[ ] Clinical data
[x]	[ ] Dual use research of concern

Methods

n/a	Involved in the study
[x]	[ ] ChIP-seq
[x]	[ ] Flow cytometry
[x]	[ ] MRI-based neuroimaging
